# The Baltimore College of Dental Surgery

**Published:** 1868-03

**Authors:** 


### EDITORIAL DEPARTMENT.

*The Baltimore College of Dental Surgery.*—The Commencement Exercises will be held on Thursday evening, March 12, at the Concordia Building to which the alumni and friends of the college are most cordially invited. Dr. Noel, Prof. of Physiology will deliver the valedictory address. Quite a number of the alumni have already notified the dean of their intention to be present, and a very pleasant and happy reunion is anticipated.

It is always a pleasure to the members of the Faculty to receive visits from the alumni and friends of the Baltimore College, and they trust that none may absent themselves on account of not receiving invitations.

Cards of invitation are annually sent to the P. O. address of all the alumni, where such is known, and the dean would be pleased to learn of any change of location, in order that invitations and circulars may be properly directed.

The number of matriculants at the Baltimore College this session is sixty-nine, which we learn from reliable sources, is thirty more than at any other dental college.

This evidence of prosperity will be as gratifying to the friends of the institution as it is encouraging to the faculty, the members of which will do all in their power to sustain the reputation which this, the first established, Dental College has always maintained.

